# Effect of interfacial and edge roughness on magnetoelectric control of Co/Ni microdisks on PMN-PT(011)

**DOI:** 10.1038/s41598-022-06285-6

**Published:** 2022-03-10

**Authors:** Y. Hsiao, D. B. Gopman, K. Mohanchandra, P. Shirazi, C. S. Lynch

**Affiliations:** 1 Department of Mechanical and Aerospace Engineering, University of California, Los Angeles, CA USA; 2 Materials Science & Engineering Division, National Institute of Standards and Technology, Gaithersburg, MD USA; 3 Bourns College of Engineering, University California, Riverside, CA USA

**Keywords:** Materials science, Engineering

## Abstract

Uniform magnetic behavior within arrays of magnetoelectric heterostructures is important for the development of reliable strain-mediated microdevices. Multiple mechanisms may contribute to observed nonuniform magnetization reversal including surface roughness, non-uniform strain, and fabrication induced imperfections. Here, Co/Ni microdisks of 7 µm diameter were produced on both [Pb(Mg_1/3_Nb_2/3_)O_3_]_1−*x*_–[PbTiO_3_]_*x*_ with *x* = 0.3 nominal composition (PMN-30PT) (011) and Si substrates, and the out-of-plane magnetization reversal was characterized using magneto-optical Kerr effect (MOKE). Coercivity variation across the microdisks within the arrays was observed on both the PMN-30PT and Si specimens with zero electric field applied. Co/Ni microdisks on a PMN-30PT substrate displayed relatively larger coercivity than those on a Si substrate due to the surface roughness effect. Quasistatic electric fields of varying magnitude were applied to the PMN-30PT substrate to assess the dependence of the coercivity on electric field induced strain. Our results indicate that while coercivity decreases with the increase of electric field induced strain, interfacial and edge roughness combine to realize a prohibitively large coercivity to overcome within the Co/Ni microdisks as well as a broad distribution of coercive field across a patterned microdisk array.

## Introduction

Strain-mediated magnetoelectric (ME) heterostructures consisting of ferromagnetic (FM) and ferroelectric (FE) constituent layers are being explored as an advantageous device structure for local control of polarization, strain and magnetization at micron and sub-micron length scales^1–3^. Small-scale magnetic structures have certain applications, such as cell-sorting^4^, cancer-cell destruction^5^, bacteria isolation^6^, and microsurgery^7^, that would realize advances in overall size, energy efficiency and precision by migrating from traditional coil-based techniques to strain-modulated magnetization in such coupled FM–FE heterostructures^8^. Uniform magnetic behavior among these ME heterostructures is critical to the large-scale manufacturability of reliable strain-mediated devices^9,10^.

In ME heterostructures, the magnetization of an overlaid thin film can be manipulated using strain from a FE substrate via magneto-elastic coupling. Single-crystal, relaxor ferroelectric (011)-oriented [Pb(Mg_1/3_Nb_2/3_)O_3_]_1−*x*_–[PbTiO_3_]_*x*_ (PMN-PT) has been investigated in prior studies for its in-plane anisotropic strain, i.e. compressive strain in the [100] direction and tensile strain along the [01−1] direction. One recent study pointed to micrometer-scale variation in the strain generated from the FE PMN-PT substrate limiting the degree of uniform remagnetization behavior in an overlaid FM^11^. However, this inhomogeneous strain distribution may not be the only contribution to non-uniform behavior. Other possible contributors include interfacial and edge roughness^12–16^. Prototype ME heterostructures were designed and fabricated to better quantify these alternative contributors to non-uniform magnetization reversal behavior. Co/Ni microdisk heterostructures were patterned on PMN-30PT (011) and Si substrates to evaluate the magnetic coercive field and its variation across substrates and under strains from applied electric fields to the FE substrate.

Co/Ni multilayers exhibit strong perpendicular magnetic anisotropy (PMA)^17–19^ energy that arises from the surfaces and interfaces of ultrathin Co and Ni layers, with each alternating layer spanning less than 1 nm^20^. As interfacial effects predominate the magnetic anisotropy energy in Co/Ni, the magneto-elastic coupling in Co/Ni is also predominantly originating from surfaces and interfaces. Co/Ni films on PMN-30PT substrates have been shown to display a larger interfacial magnetostriction than the volume contribution^21^. This work was designed to explore the distribution of magnetic behavior across arrays of Co/Ni microdisks on a given substrate and to clarify the relative contributions of surface roughness, strain, and processing-induced lateral inhomogeneity that arises in the fabrication process. The coercivity of each Co/Ni microdisk was measured using magneto-optical Kerr effect (MOKE) magnetometry. Atomic force microscopy (AFM) was used to characterize the surface roughness of the Co/Ni multilayers on PMN-30PT and Si substrates. Scanning electron microscopy (SEM) was used to investigate the lateral inhomogeneity of patterned Co/Ni microdisks. The coercive field distribution was measured in an unstrained (zero electric field) Co/Ni microdisk array grown on PMN-30PT and compared to an identical array grown on a smooth Si wafer to observe how the surface roughness affected the coercive field and the coercive field distribution across each array. The distribution of the coercive field among the heterostructures fabricated on the smooth Si is attributed to variations introduced in the fabrication process. The difference in the median coercive field between the smoother Si and rougher PMN-30PT specimens was attributed to surface roughness. Application of an electric field was used to study the influence of strain on the coercive field response in the magnetostrictive Co/Ni heterostructures.

## Experimental section

### Fabrication of Co/Ni heterostructures

**Figure 1:**
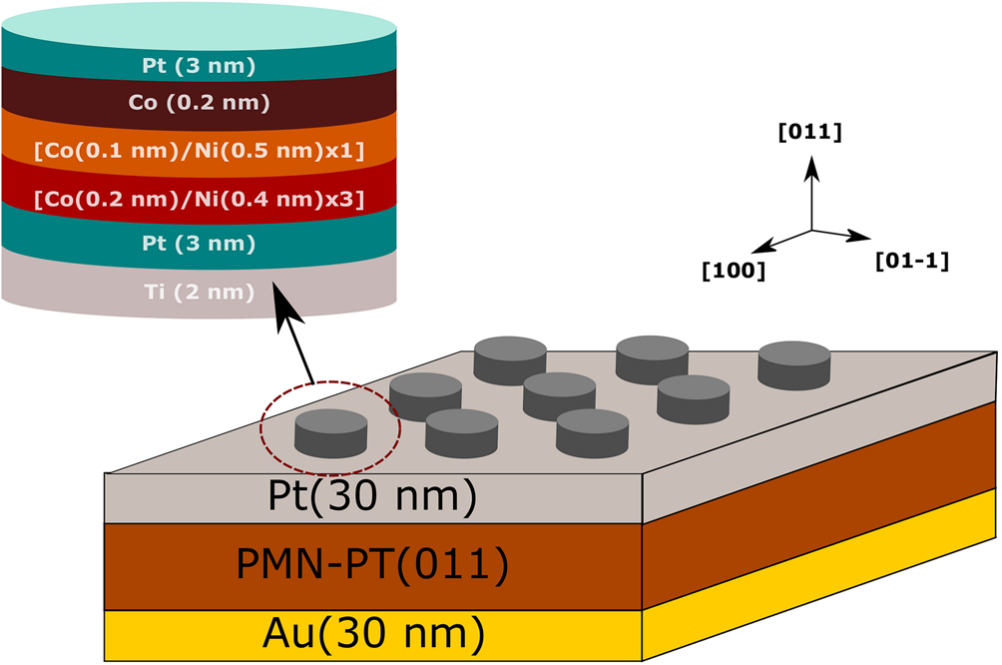
The structure of Co/Ni microdisks on the PMN-30PT substrate.

Co/Ni microdisks were patterned on a 500 µm thick PMN-30PT substrate (011) single crystal (TRS Technologies, Inc., United States) as shown in Fig. 1 (Certain commercial equipment, instruments, or materials are identified in this paper in order to specify the experimental procedure adequately. Such identification is not intended to imply recommendation or endorsement by NIST, nor is it intended to imply.). As depicted from the coordinate axes of Fig. 1, the Co/Ni disks were deposited over the (011)-cut PMN-PT substrate, for which applied electric fields generate substantial expansion along the 01–1 axis with a more modest compression along the 100 axis. Electric-field induced strains are transferred to the films clamped to the substrate to induce changes in the Co/Ni magnetization.

Electron beam evaporation was used for the deposition of Ti, Pt, Co, Ni, and Au at room temperature at a base pressure 3 × 10^–4^ Pa (2 × 10^–6^ Torr). The top and bottom surfaces of the PMN-30PT substrate were coated with 30 nm thick Au and Pt electrodes, respectively. The PMN-30PT with the electrodes was poled along the [011] direction with an electric field of 0.8 MV/m across the substrate for approximately one minute.

The poled PMN-30PT substrate was cleaned using acetone, methanol, isopropanol, and a one-minute oxygen plasma treatment (80 W radio frequency power, 500 Pa, 50 °C) prior to the deposition of the Co/Ni films. A 2 nm thick Ti film was evaporated on the Pt electrode. Using a known recipe that consistently delivers high perpendicular magnetic anisotropy, we employ here three repeated Co(0.2 nm)/Ni(0.4 nm) bilayers followed by a Co(0.1 nm)/Ni(0.5 nm) bilayer were grown on the Pt(2 nm) film. Pt was selected as a seed layer to enhance the PMA by promoting face-centered-cubic (111)-textured growth of the Co/Ni films^22,23^. Although certain layers have attributed thicknesses below a single lattice spacing, it is understood that this refers to a fractional monolayer coverage by that particular layer. The nominal layer thickness of Co and Ni were obtained using a 6 MHz quartz crystal thickness monitor (Inficon, Inc., Switzerland). A Co (0.2 nm) layer was added for symmetry and capped with 3 nm thick Pt layer to prevent metal oxidation. The films were patterned into microdisks of 7 µm diameter by a lift-off technique using nLof2020 photoresist (MicroChemicals GmbH, Wiesbaden, Germany). Following the same procedure, microdisks with identical 7 µm diameter were patterned on the 500 µm thick single crystalline Si (001) substrate without the electrodes to assess possible effects of the substrate roughness.

### Characterization methods

#### Surface roughness

A BRUKER ICON (Bruker, Goleta, CA) atomic force microscope (AFM) was used to measure the surface roughness of the Pt film on the PMN-30PT and Si substrates by scanning regions of lateral extent 20 μm × 20 μm and 10 μm × 10 μm respectively at 1 Hz in tapping mode. A CoCr-coated AFM tip with a resonance frequency of 75 kHz was used for imaging. The average arithmetic roughness (*R*_*a*_) was calculated from 2.5 × 2.5 μm area at five different locations on the flattened image.

#### Shape variations in Co/Ni microdisks

A FEI Nova 230 scanning electron microscope was used to visualize the shape variation in Co/Ni microdisks. Images were collected using an acceleration voltage of 3 kV following a working distance of 5.7 mm. The ImageJ^24^ software was used to determine the perimeter and area of 63 microdisks. The microdisk’s circularity (*C*) was calculated by the following Eq. (1),

1 $$C=\frac{4\pi *Area}{(Perimete{r)}^{2}}$$

#### In-plane strain

Two axial strain gauges with a gauge factor of 1.51 and gauge resistance of 120 Ω (Omega Engineering Inc.) were used to measure electric field-induced strain in the PMN-PT substrate. The change in the strain gauge resistance was monitored using an amplified signal from a Wheatstone bridge and recorded by an analog to digital converter (NI DAQ with NI-9237 module in a quarter bridge configuration). The strain gauges were bonded to the PMN-30PT substrates with top and bottom electrodes and excited with a voltage of 375 V to generate an electric field of 0.75 MV/m.

#### Magnetization and magnetic anisotropy

A superconducting quantum interference device (SQUID) magnetometer was used to determine the in-plane and out-of-plane magnetization of the films on PMN-PT. SQUID magnetometry was performed using a Quantum Design MPMS©3 SQUID magnetometer at 298 K. The in-plane and out-of-plane magnetizations were determined by sweeping the magnetic field from − 1 to 1 T.

#### Magnetic coercive field

A magneto-optic Kerr effect (MOKE) system was used to visualize the magnetization reversal process in the Co/Ni microdisk arrays on both the PMN-30PT and Si substrates. The magnetic field was applied perpendicular to the heterostructures while monitoring the real-time magnetization reversal process. Magnetic domain imaging was carried out using a Leitz Orthoplan polarizing microscope in reflection mode, monitored by a 4 Megapixel CCD camera (Thorlabs 4070M-USB). One full cycle of the recorded video was 100 s. The frame rate for video recording was 12 frames per second with sweeping rate 1.85 mT/s for the PMN-30PT samples. Three frames were taken per second for the Si samples with sweeping rate 1.99 mT/s. The range of the applied magnetic field was − 46.20 mT to 46.20 mT with an interval 0.16 mT for the microdisks on the PMN-30PT substrate, and from − 49.79 mT to 49.79 mT with the interval of 0.67 mT for the microdisks on the Si substrate, respectively. The measurement was performed with five values of electric field, {0, 0.2, 0.4, 0.6, 0.8} MV/m, applied to the substrate.

**Figure 2:**
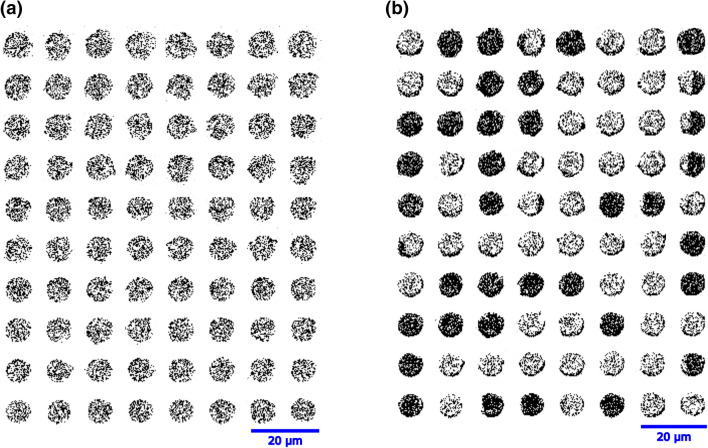
MOKE imaging of the microdisks on the PMN-30PT substrate under the magnetic fields (**a**) 0 mT, (**b**) 37.8 mT. The light and dark contrast corresponds to the magnetization pointing up and down with respect to the substrate surface.

**Figure 3:**
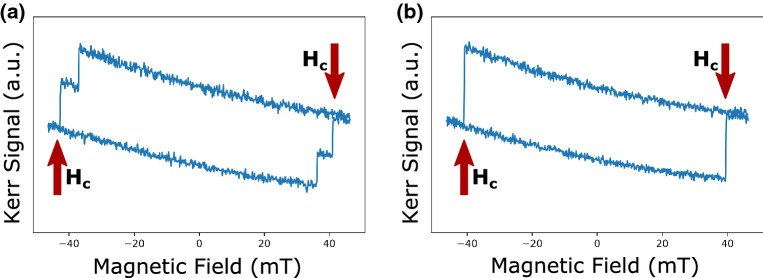
Illustration of a MOKE hysteresis loop with (**a**) multiple jumps (**b**) single jump.

Figure 2a and b show the MOKE images of the magnetization reversal under a time varying magnetic field used to obtain the coercive field value of the individual microdisks. The *μ*_*0*_*H*_*C*_ of the individual Co/Ni microdisks were obtained from images extracted from the video with the applied magnetic field at different magnitudes. A curve of the contrast intensity (i.e. Kerr intensity) as a function of the applied magnetic field was generated for each microdisk. The magnetization reversal was often accompanied by more than one discrete remagnetization jump, indicating significant domain wall pinning during reversal. When the hysteresis loop of a microdisk displayed more than one magnetization jump during reversal, the final jump in the magnetization within a transition (e.g. from up-to-down) was attributed to the coercive field (*μ*_*0*_*H*_*C*_) value for the microdisk as depicted in Fig. 3a and b. The slope observed in the hysteresis loop is due to the Faraday effect and does not affect the coercive field measurement. The median of *μ*_*0*_*H*_*C*_ was calculated from the measured μ_0_*H*_C_ values of the microdisks on the PMN-30PT and the Si substrates.

## Results and discussion

### Contributors to non-uniform magnetization switching behaviors on PMN-30PT

**Figure 4:**
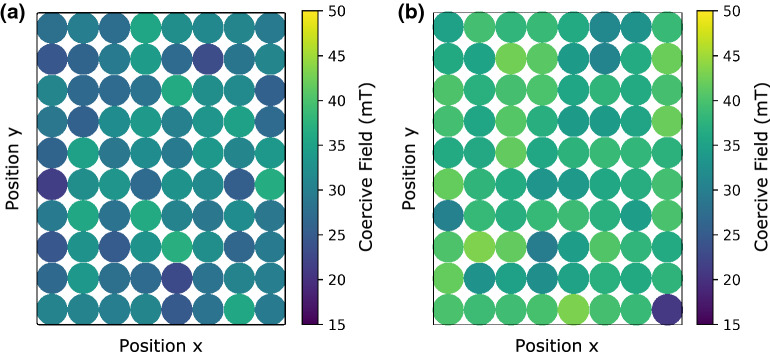
The coercive field distribution of an 8 by 10 array of microdisks on (**a**) Si and (**b**) PMN-30PT substrates. Each circle represents a microdisk.

A contour map was created to visualize the average coercive field of each disk to understand the variation in the coercive field among the microdisks. The results are shown in Fig. 4a and b for the eighty microdisks on the PMN-30PT substrates, respectively. The data on PMN-30PT were acquired in the absence of an electric field. Lateral inhomogeneity in the coercivity of Co/Ni microdisks is observed in both cases. The μ_0_*H*_C_ median for the Co/Ni microdisks on the PMN-30PT substrate was found to be 37.31 mT, whereas the μ_0_*H*_C_ median for the Co/Ni on the Si substrate was 29.95 mT, more than twenty percent lower in magnitude.

In prior work, the laterally non-uniform behavior of the magnetization reorientation of heterostructures on a PMN-30PT substrate was attributed to local strain variations arising from the domain structure in the PMN-30PT substrate^11^. However, here we have additionally demonstrated μ_0_*H*_C_ variations across the arrays of the Co/Ni microdisks on both PMN-30PT and Si substrates, where the Si substrate does not possess local variations in strain and has significantly lower surface roughness. This indicates that there are additional contributions to the variations in the coercive field across the microdisk arrays. Surface roughness of the substrate and lateral inhomogeneity of the Co/Ni microdisks appear to be the most significant contributors to the non-uniform μ_0_*H*_C_ distribution.

Recent studies have shown that the coercivity of deposited films is affected by the surface roughness of the substrate^14,15,25^. In evaporated layered structures like ultrathin Co/Ni multilayers, surface roughness is particularly significant as the perpendicular magnetic anisotropy and coercivity are strongly sensitive to the interfaces between alternating layers. Indeed, the broken symmetry at the Pt/Co and Co/Ni interfaces is key to a sizable asymmetry in orbital moment occupation^26^ which in turn leads to a strong spin–orbit interaction key to the out-of-plane magnetization direction. Deviations from a perfectly abrupt interface between layers will both reduce the average magnetic anisotropy energy and also lead to local variation in the coercive field. The average *R*_a_ of Co/Ni microdisks on PMN-30PT substrate was (2.8 ± 0.4) nm and that of Pt film on PMN-30PT was (2.2 ± 0.3) nm. Both are significantly larger than that of Co/Ni film on Si, which was measured as (0.6 ± 0.1) nm. Uncertainties reflect the one sigma variance of the estimated arithmetic roughness. The higher roughness of the PMN-30PT surface produces a higher local depinning field^15^, defined as the external magnetic field required to move domain walls from pinning sites. The nearly 25% increase in coercivity for Co/Ni microdisks on PMN-30PT likely implies a larger depinning field for domain walls than for microdisks grown directly on a Si substrate.

Lateral inhomogeneity in the coercivity of Co/Ni microdisks and roughness due to substrate choice were addressed in the previous paragraph. In order to understand the inhomogeneity caused by micromanufacturing, the dimensions of the 63 Co/Ni microdisks were measured by SEM. The average circularity of the microdisks was (0.18 ± 0.05), which is particularly low and is a strong deviation from the nominal circular disk shape that was intended for the patterned disks. Uncertainty reflects the one sigma variance of the estimated circularity across over 63 microdisks. The nearly 30% uncertainty of average circularity implies that variations and imperfections in the microfabrication process aids to the non-uniform *μ*_*0*_*H*_*C*_ distribution observed in the Co/Ni microdisks.

### Strain effect on the coercivity of the Co/Ni microdisks

**Figure 5:**
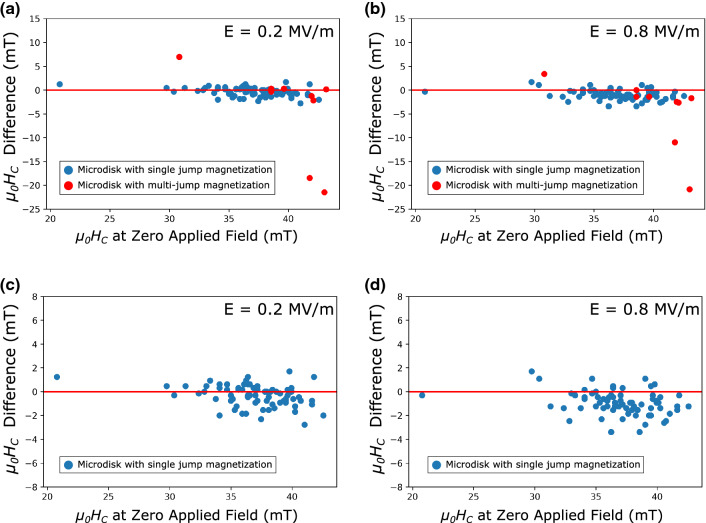
Scatter plots of μ_0_*H*_C_ at zero applied E-field versus the μ_0_*H*_C_ difference under the applied electric fields (**a**) between 0 and 0.2 MV/m (**b**) between 0 and 0.8 MV/m. (**c**) and (**d**) represent the microdisks with a single μ_0_*H*_C_ value in (**a**) and (**b**), respectively.

Now that the underlying factors contributing to the non-uniform variation in the coercive field of the microdisks on PMN-30PT have been articulated, the magnetoelectric performance of the microdisks is examined. Figure 5a and b show the change of μ_0_*H*_C_ correlated to the initial μ_0_*H*_C_ at zero applied electric Supplementary Informationfield for the entire family of 80 microdisks under electric fields of 0.2 MV/m and 0.8 MV/m respectively. The corresponding strain values of the applied field to the PMN-30PT substrate can be found in the . The red data points represent microdisks that exhibited multiple jumps during the magnetization reversal process as seen in Fig. 3a. Figure 5c and d shows the microdisks with single-jump magnetization reversal processes denoted by blue dots to distinguish the coercive field distribution under the application of increasing electric fields. After removing the red data points, it remains evident that the coercive field distribution across the microdisks is shifted towards lower *μ*_*0*_*H*_*C*_ values as the magnitude of the applied electric field was increased from 0.2 to 0.8 MV/m, signifying the magnetoelectric effect on microdisk reversal.

**Figure 6:**
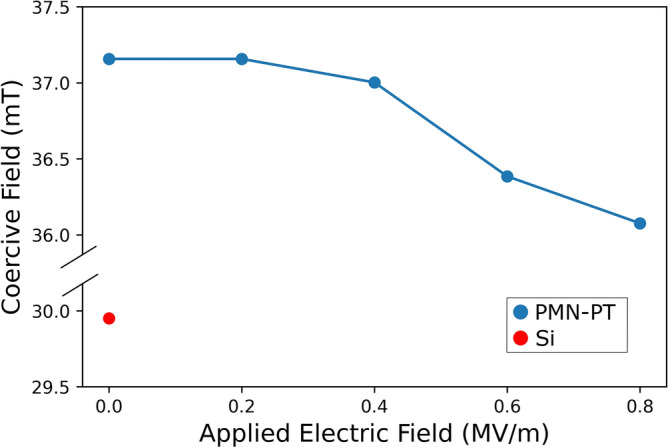
Median coercive field as a function of the applied electric field to the PMN-30PT and Si substrates. Line drawn to aid reader’s eyes.

Figure 6 shows the calculated *μ*_*0*_*H*_*C*_ median for the microdisks with single jump magnetization reversal versus the external applied electric field on the PMN-30PT substrate. The trend in Fig. 6 reflects the magnetoelectric coupling between the PMN-30PT substrate and the magnetostrictive Co/Ni microdisks. As the electric field-induced strain increases, the average coercive field of the Co/Ni microdisk array decreases due to the change in magnetoelastic energy. This corresponds to a reduction in the energy barrier for magnetization reversal, and a reduction in the perpendicular anisotropy energy. The weakened PMA is consistent with the strain-induced change in the magnetoelastic energy, and interfacial anisotropy modulation via strain has been observed in other Co-based multilayers^27^. However, the largest change in *μ*_*0*_*H*_*C*_ under an electric field is less than the difference between the coercive fields of the magnetic structures on the Si substrate and the PMN-30PT substrate at zero applied electric field. The *μ*_*0*_*H*_*C*_ median of Co/Ni microdisks on the Si substrate shows smaller coercivity than that of Co/Ni microdisks on the PMN-30PT substrate under each applied electric field. This implies the surface roughness effect is larger than the magnetoelastic effect on the coercivity of the patterned microdisks.

The standard deviations (SDs) of *μ*_*0*_*H*_*C*_ at a zero field were used as a comparison of the coercive field dispersion across the arrays on both PMN-30PT and Si substrates. The *μ*_*0*_*H*_*C*_ standard deviations of the microdisks on PMN-30PT substrate were [3.34, 3.15, 3.31, 3.29, 3.19] mT for the applied field [0, 0.2, 0.4, 0.6, 0.8] MV/m respectively, and that on Si substrate was found to be 3.46 mT. The coercivity standard deviation values of 3.34 mT and 3.46 mT were found for Co/Ni microdisks on the PMN-30PT under zero applied field and Si respectively. The difference among the standard deviations for each of the applied fields were less than 0.2 mT. The values of *μ*_*0*_*H*_*C*_ dispersions do not show strong correlation with either the substrate roughness or the strain. The difference of the *μ*_*0*_*H*_*C*_ dispersions are more likely a consequence of edge roughness/circularity variation. The results indicate that surface roughness and strain shift the coercivity but do not affect the *μ*_*0*_*H*_*C*_ dispersions significantly.

### Magnetoelastic energy discussion

**Figure 7:**
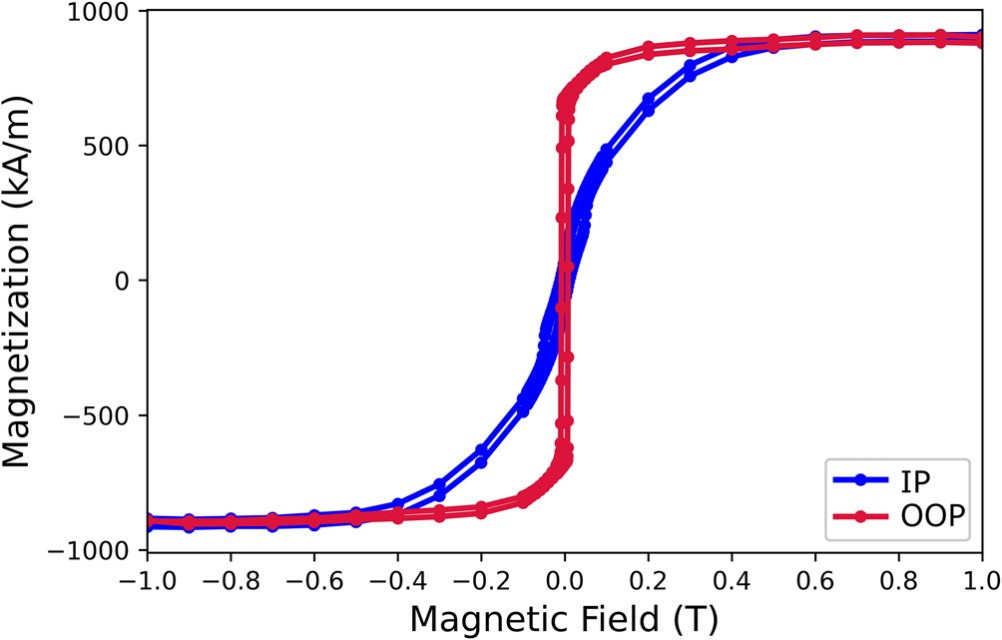
Hysteresis loop of Co/Ni films for both in-plane (IP) and out-of-plane (OOP) directions.

In this section, magnitude of magnetoelastic energy density is compared with anisotropy energy density to understand the strain-induced coercivity. SQUID measurements were performed to obtain anisotropy field (μ_0_*H*_k_) and saturation magnetization (*M*_*s*_). Figure 7 shows the hysteresis loop of Co/Ni films of both in-plane (IP) and out-of-plane (OOP) directions from the SQUID measurements. The results indicate easy axis out-of-plane. The μ_0_*H*_k_ was measured to be 0.4 T and the *M*_*s*_ was found to be 910 kA/m.

PMA Co/Ni films exhibit uniaxial anisotropy. The effective anisotropy energy density for the Co/Ni films can be expressed by Eq. (2)

2 $${E}_{anistropy}=\frac{1}{2}{\mu }_{0}{M}_{S}{H}_{K}{\mathrm{sin}}^{2}\theta $$

where ${\mu }_{0}$ is permeability of free space; $\theta$ is the polar angle relative the orientation normal to the sample. *M*_s_ and ${\mu }_{0}$ *H*_k_ were estimated as 910 kA/m and 0.4 T from Fig. 7.

The magnetoelastic energy density change was determined using the following equations^27^:

3 $$\Delta {E}_{me}=\frac{{B}_{1}}{6}\left({\varepsilon }_{x}-{\varepsilon }_{y}\right)+\frac{{B}_{2}}{6}[5{\varepsilon }_{x}+{\varepsilon }_{y}+\frac{6\nu }{1-\nu }\times ({\varepsilon }_{x}+{\varepsilon }_{y})]-\frac{{B}_{1}+2{B}_{2}}{3} ({\varepsilon }_{x}-{\varepsilon }_{y})$$

where ${\varepsilon }_{x}$ and ${\varepsilon }_{y}$ are in-plane strains in [100] and [01−1] directions. ${\varepsilon }_{x}$ =-1500 µ and ${\varepsilon }_{y}$ = 500 µ under the field 0.8 MV/m can be found in Supplementary Information. Poisson’s ratio $\nu$ = 0.3. ${B}_{1}$ and ${B}_{2}$ are expressed as a function of magnetostriction (${\lambda }_{100}$, ${\lambda }_{111}$) and elastic constants (${c}_{11}$, ${c}_{12}$, ${c}_{14}$) by Eqs. (4) and (5)

4 $${B}_{1}=-\frac{3}{2}{\lambda }_{100}\left({c}_{11}-{c}_{12}\right)$$

5 $${B}_{2}=-3{c}_{44}{\lambda }_{111}$$

Lacking empirical data on the nanoscale mechanical properties of Ni or Co, the approximation of using bulk literature values was made. Given that Co and Ni have comparable values in their elastic constants, and the multilayer films behave structurally pseudomorphic^28^, bulk values of Ni (c_11_ = 250 GPa, c_12_ = 160 GPa, c_44_ = 118.5 GPa) were used in the approximation of B_1_ and B_2._ Magnetostriction in Co/X multilayers^29^, where X = Pt, Cu, Ag, Au has been observed with the order of magnitude ${10}^{-4}$. This value was used for the estimated value of ${\lambda }_{100}$ and ${\lambda }_{111}$. ${E}_{anistropy}$ was estimated to be on the order of 100 kJ/m^3^, which is significantly larger than the contribution of magnetoelastic energy density (less than 10 kJ/m^3^) induced by the applied electric field. This limited the total effect of magnetoelectric modulation on the Co/Ni microdisks, particularly with respect to engineering any significant rotation of the Co/Ni magnetization away from its out-of-plane easy axis. This is also consistent with the modest strain-induced 2.9% reduction of coercive field at 0.8 MV/m electric field in the PMN-30PT substrate.

## Conclusion

In this study, Co/Ni microdisks were fabricated on PMN-30PT (011) and Si substrates and the effect of surface roughness, in-plane strain, and manufacturing defects were assessed. *μ*_*0*_*H*_*C*_ variations across the Co/Ni microdisk arrays were observed on both the Si and PMN-30PT substrates with zero applied field, and the standard deviations of the *μ*_*0*_*H*_*C*_ distribution were comparable for both arrays showing that surface roughness was not primary cause of the observed dispersion in *μ*_*0*_*H*_*C*_. This suggests that the *μ*_*0*_*H*_*C*_ variation was more likely the result of variations introduced in the fabrication process. The *μ*_*0*_*H*_*C*_ of Co/Ni microdisks on the PMN-30PT substrate was larger than that on the Si substrate by nearly 25%. The larger *μ*_*0*_*H*_*C*_ observed for the Co/Ni microdisks on the PMN-30PT specimen was attributed to an increase in the depinning field. Coercivity was measured with different applied electric field to the PMN-30PT. The results indicate that strain induced by the electric field lowers the *μ*_*0*_*H*_*C*_ of Co/Ni. The strain induced modulation of the coercivity is modest when compared to the contributions from surface roughness and patterning-induced disorder. The estimated anisotropy energy density was larger than the strain-induced energy density change with an order of magnitude difference. This also explains the limited strain effect on the modulation of coercivity. Design of strain modulated PMA based devices will require selection of material systems and processing technique that lead to lower interfacial roughness, and improved edge/sidewall uniformity of microstructures, in order to realize a smaller coercivity for strain induced coercive field modulation.

## Supplementary Information

Supplementary Information.
