# Supplementary material for: Effect of interfacial and edge roughness on magnetoelectric control of Co/Ni microdisks on PMN-PT(011)
Source: Sci Rep. 2022 Mar 10;12:3919. doi: 10.1038/s41598-022-06285-6 (PMC8913801; doi:10.1038/s41598-022-06285-6)
Supplement: Supplementary file 1 — Supplementary Information. [file 41598_2022_6285_MOESM1_ESM.pdf]

Effect of Interfacial and Edge Roughness on Magnetoelectric Control of Co/Ni Microdisks on PMN-PT(011)

Y. Hsiao[1], D. B. Gopman[2] , K. Mohanchandra[1], P. Shirazi[1], C. S. Lynch[1,3]
1. Department of Mechanical and Aerospace Engineering, University of California, Los Angeles, CA
2. Materials Science & Engineering Division, National Institute of Standards and Technology, Gaithersburg, MD
3. Bourns College of Engineering, University California, Riverside, CA

Author to whom correspondence should be addressed: cslynch@engr.ucr.edu

The in-plane strain components produced in the PMN-30PT as a function of out-of-plane electric field in both [01-1] and [100] directions are shown in Figure 1.

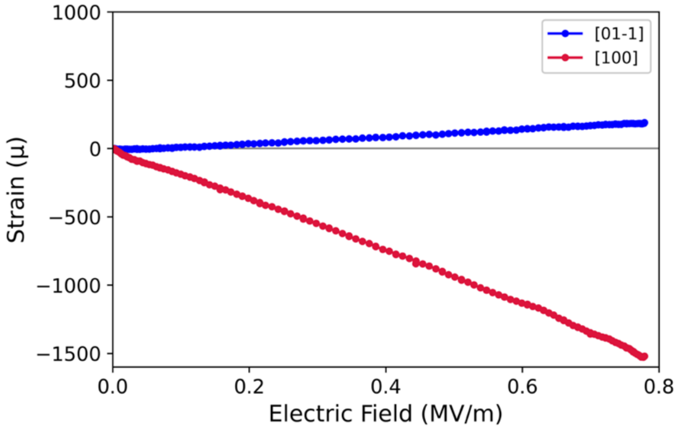

Figure S1. In-plane microstrain along the directions [01-1] and [100] as a function of the applied electric field.
